# Genotyping of *Enterocytozoon bieneusi* in Farmed Blue Foxes (*Alopex lagopus*) and Raccoon Dogs (*Nyctereutes procyonoides*) in China

**DOI:** 10.1371/journal.pone.0142611

**Published:** 2015-11-06

**Authors:** Wei Zhao, Weizhe Zhang, Ziyin Yang, Aiqin Liu, Longxian Zhang, Fengkun Yang, Rongjun Wang, Hong Ling

**Affiliations:** 1 Department of Parasitology, Harbin Medical University China; Harbin Medical University, Heilongjiang Provincial Key Laboratory for Infection and Immunity; Heilongjiang Key Laboratory for Pathogen Biology, Harbin, Heilongjiang 150081, China; 2 College of Animal Science and Veterinary Medicine, Henan Agricultural University, Zhengzhou, Henan 450002, China; The University of Hong Kong, HONG KONG

## Abstract

*Enterocytozoon bieneusi* is the most common species of microsporidia found both in humans and animals. Farmed animals, particularly closely associated to humans, may play an important role of zoonotic reservoir in transmitting this disease to humans. The fur industry is a major economic component in some parts of China. To understand the prevalence, genotype variety and zoonotic risk of *E*. *bieneusi* in farmed foxes and raccoon dogs, two species of fur animals, fecal specimens of 110 blue foxes and 49 raccoon dogs from Heilongjiang and Jilin provinces in China were examined by internal transcribed spacer (ITS)-based PCR. *E*. *bieneusi* was detected in 16.4% (18/110) blue foxes and 4.1% (2/49) raccoon dogs. Altogether, four genotypes of *E*. *bieneusi* were identified, including two known genotypes D (n = 13) and EbpC (n = 5), and two novel genotypes named as CHN-F1 (n = 1) in a fox and CHN-R1 (n = 1) in a raccoon dog. Phylogenetic analysis revealed that all the four genotypes were the members of zoonotic group 1. Genotypes D and EbpC were found in humans previously. The findings of zoonotic genotypes of *E*. *bieneusi* in the foxes and raccoon dogs suggest these animals infected with *E*. *bieneusi* may pose a threat to human health.

## Introduction

Microsporidia are a phylum of spore-forming unicellular obligate intracellular eukaryotes, which are made up of over 1300 species representing at least 160 genera [1]. They can be found in almost all the animal phyla and are ubiquitous in the environments [2]. To date, more than 14 micosporidian species have been identified in humans [3], and among them *Enterocytozoon bieneusi* is the most common species of microsporidia found in humans [4]. Clinical features of microsporidiosis caused by *E*. *bieneusi* are persistent diarrhea, abdominal pain and weight loss in immunodeficient individuals, such as persons with AIDS and organ transplant recipients; in otherwise immunocompetent or healthy individuals, *E*. *bieneusi* causes a self-limiting diarrhea, even sometimes appears asymptomatic [4,5]. Besides humans, *E*. *bieneusi* has been detected in various animal hosts, including mammals, birds, rodents and reptiles [6,7], indicating that these animals can be the potential reservoir hosts of human *E*. *bieneusi* infection. Animals infected with *E*. *bieneusi* can shed spores with feces into the environment. Most human infections of *E*. *bieneusi* are thought to result from fecal-oral transmission of spores from infected hosts through contaminated water or food [3]. Infective spores of *E*. *bieneusi* have been detected in drinking source watershed, recreational bathing water, and wastewater plants [8–11]. A food-borne outbreak of microsporidiosis caused by *E*. *bieneusi* has been reported in Sweden, which was considered to be probably associated with contaminated fresh produce served to hotel guests [12]. Current epidemiological data of *E*. *bieneusi* have raised public health concern about the zoonotic nature, and water- and food-borne transmission of *E*. *bieneusi*.

A high degree of genetic polymorphism has been observed in the internal transcribed spacer (ITS) region of the ribosomal RNA (rRNA) gene within *E*. *bieneusi*. Sequence analysis of the ITS gene is the standard method for genotyping *E*. *bieneusi* isolates in humans and animals, and a standardized nomenclature for genotypes of *E*. *bieneusi* based on ITS gene sequencing has been proposed [13]. To date, molecular analysis of the ITS gene of *E*. *bieneusi* has enabled division of this pathogen into over 240 genotypes found in a wide range of host species including humans [4,6,14]. At present, humans have been reported to carry 64 genotypes with 33 of them being found in animals, suggesting the potential of zoonotic transmission of *E*. *bieneusi* [4,15]. By phylogenetic analysis of the ITS gene sequences, all the genotypes of *E*. *bieneusi* are divided into eight different groups [16]. A large cluster named as group 1 contains more than 94% published genotypes of *E*. *bieneusi* [17]. In this group, some genotypes are found both in humans and animals, and although some genotypes are currently found only in animals, they show a close genetic relationship to human-pathogenic genotypes, thus, establishing the zoonotic potential of the genotypes in group 1 [17]. The remaining groups, named as group 2 to group 8 are found mostly in specific hosts and wastewater [10]. To improve our understanding of the epidemiology of human microsporidiosis and strengthen the study of *E*. *bieneusi* populations as well as assessment of the role of animals in the transmission of this pathogen to humans, epidemiological surveys should focus on genotyping *E*. *bieneusi* isolates from under-sampled hosts [18].

Currently, very limited genetic data are available on fox- and raccoon dog-harboring *E*. *bieneusi*. To date, only two studies described the occurrence of *E*. *bieneusi* in wild foxes [19,20] and there is no report concerning *E*. *bieneusi* infection in raccoon dogs. It is well-known that farmed foxes and raccoon dogs as fur animals, are more closely associated to humans than wild ones. However, the prevalence and genetic characteristics of *E*. *bieneusi* in these animals are still unknown. In China, there are approximately 3.44 million farmed foxes and 3.5 million farmed raccoon dogs, and farming of fur animals represents an important endemic income in some parts/areas. Blue foxes (*Alopex lagopus*) and raccoon dogs (*Nyctereutes procyonoides*) are common fur animal species farmed in Heilongjiang and Jilin provinces in the northeast of China. Considering that *E*. *bieneusi* has been found in humans and many kinds of animals in the investigated areas, including pigs, cattle, sheep, goats, deer, dogs, cats and a chicken [14,21,22–26], in the present study, to understand the prevalence of *E*. *bieneusi* and genetic structure of parasite populations circulating among these animals, *E*. *bieneusi* isolates in blue foxes and raccoon dogs were identified and genotyped by PCR and sequence analysis of the ITS region of the ribosomal gene. Zoonotic potential of *E*. *bieneusi* genotypes was assessed, and the genetic diversity of novel *E*. *bieneusi* genotypes and their relationship with known ones were presented by constructing a neighboring-joining tree.

## Materials and Methods

### Ethics statement

The present study protocol was reviewed and approved by the Research Ethics Committee and the Animal Ethical Committee of Harbin Medical University. All work with animals strictly followed guidelines in accordance with the Regulations for the Administration of Affairs Concerning Experimental Animals. Prior to fecal specimen collection, we contacted the animal owners or managers and obtained their permission. During the procedure of collecting specimens, we did not do harm to these animals involved in the present study at all.

### Specimen collection

During the period from June 2014 to March 2015, a total of 159 fecal samples were collected from 110 blue foxes and 49 raccoon dogs on five farms (S1 Text) located in Heilongjiang and Jilin provinces, China (Table 1) (S1 Fig). Farms were selected only based on the owners’ willingness to participate and the accessibility of animals for sampling. Fresh fecal specimen of each animal was collected from the bottom of cages on the dishes below the wire net bottom immediately after defecation by using a sterile disposal latex glove and then placed into an individual plastic bag. All the fecal specimens were transported to the laboratory in a cooler with ice packs within 24 hours. The number of collected specimens accounted for approximately 10% of total animals in each farm. All the animals were seven or eight years old and had no apparent clinical signs of illness at the time of sampling.

**Table 1:** Prevalence and genotypes of *E*. *bieneusi* in blue foxes and raccoon dogs.

Animal species	Farm	Geographical origins (province)	No. of positive/ no. of examined (%)	Genotype (n)
Blue fox	Farm 1	Bayan (Heilongjiang)	6/50 (12.0%)	D (6)
	Farm 2	Suihua (Heilongjiang)	6/23 (26.1%)	CHN-F1 (1), EbpC (5)
	Farm 3	Changchun (Jilin)	2/16 (12.5%)	D (2)
	Farm 4	Changchun (Jilin)	4/21 (19.1%)	D (4)
	Subtotal		18/110 (16.4%)	D (12), EbpC (5), CHN-F1 (1)
Raccoon dog	Farm 5	Bayan (Heilongjiang)	2/49 (4.1%)	D (1), CHN-R1 (1)
Total			20/159 (12.6%)	D (13), EbpC (5), CHN-F1(1), CHN-R1(1)

### DNA extraction

Prior to DNA extraction, all the fecal specimens were processed by sieving and concentrating as well as washing three times with distilled water by centrifugation for 10 minutes at 1500 g. Genomic DNA was extracted from approximately 200 mg processed specimen using a QIAamp DNA Stool Mini Kit (QIAgen, Hilden, Germany) according to manufacturer-recommended procedures. To obtain high yield of DNA, the lysis temperature was increased to 95°C according to the manufacturer’s suggestions. DNA was eluted in 200 μl of AE and stored at −20°C in a freezer prior to PCR analysis.

### PCR amplification

All the DNA preparations were detected for the presence of *E*. *bieneusi* by nested PCR amplification of a 389 bp nucleotide fragment of the rRNA gene of *E*. *bieneusi* containing 76 bp of the 3’-end of SSU rRNA gene, 243 bp of the ITS region and 70 bp of 5’-region of LSU rRNA gene, and the primers and the cycling parameters in nested PCR analysis were used as previously described by Buckholt et al [27]. TaKaRa Taq DNA Polymerase (TaKaRa Bio Inc., Tokyo, Japan) was used for all the PCR amplifications. A negative control with no DNA added was included in all PCR tests. All the secondary PCR products were subjected to electrophoresis in a 1.5% agarose gel and visualized by staining the gel with ethidium bromide.

### Nucleotide Sequencing

All the secondary PCR products of the anticipated size were directly sequenced with a set of primers used for the secondary PCR on an ABI PRISM 3730 XL DNA Analyzer by Sinogeno- max Biotechnology Co. Ltd. (Beijing, China), using the Big Dye Terminator v3.1 Cycle Sequencing Kit (Applied Biosystems, USA). Sequence accuracy was confirmed by two-directional sequencing and by sequencing a new PCR product if necessary for some DNA preparations.

### Sequence analysis

Nucleotide sequences obtained in the present study were aligned with each other and reference sequences downloaded from GenBank database by Clustal X 1.83 (http://www.clustal.org/) to determine *E*. *bieneusi* genotypes. The genotypes obtained here were given the first published name if they were identical to known genotypes in GenBank. In contrast, the genotypes with single nucleotide substitutions, deletions or insertions compared to the known genotypes were considered novel genotypes and would be given new names. Of course they all needed to obtain a further confirmation by DNA sequencing of at least two PCR products. Genotyping was based on 243 bp long ITS region of the ribosomal gene according to the established nomenclature system [13].

### Phylogenetic analysis

All the aligned ITS gene of *E*. *bieneusi* sequences were implemented in the software Mega 5 (http://www.megasoftware.net/) to construct a neighbor-joining tree rooted with GenBank sequence DQ885585, based on the evolutionary distances calculated by a Kimura 2-parameter model. The reliability of these trees was assessed using bootstrap analysis with 1000 replicates.

## Results

### Prevalence of *E*. *bieneusi* in blue foxes and raccoon dogs

E. bieneusi was detected in 12.6% (20/159) animal specimens by PCR amplifying the ITS gene of E. bieneusi (S1 Table). The infection rate of E. bieneusi in blue foxes (16.4%, 18/110) was higher than that in raccoon dogs (4.1%, 2/49) (Table 1). For blue foxes, the highest infection rate of E. bieneusi was observed in Farm 2 in Suihua (26.1%; 6/23), followed by Farm 4 in Changchun (19.1%; 4/21), Farm 3 in Changchun (12.5%; 2/16) and Farm 1 in Bayan (12.0%; 6/50) (Table 1).

### Genetic characterizations and genotype distribution of *E*. *bieneusi* in blue foxes and raccoon dogs

All the 20 PCR-positive products were sequenced successfully and the genotypes were determined. Nucleotide sequences obtained in the present study were compared with those in the GenBank database by BLAST analysis. Sequence analysis revealed the presence of four different genotypes, including two known genotypes—D (n = 13) and EbpC (n = 5)—and two novel genotypes named as CHN-F1 (n = 1) and CHN-R1 (n = 1) [GenBank: KR998501 and KR998502]. Genotypes CHN-F1 and CHN-R1 differed from genotypes CHN-DC1 and Peru6 in only one base in the ITS region, respectively. Genotype D could be found in 65.0% (13/20) animals including 12 blue foxes and one raccoon dog, and it appeared in four out of five farms, showing a predominance in these animals and a wide farm distribution. Genotype EbpC was only found in five blue foxes of Farm 2, while genotypes CHN-F1 and CHN-R1 were only found in one fox at Farm 2 and one raccoon dog at Farm 5, respectively (Table 1). No mixed infections were identified in these animals in the present study.

### Phylogenetic relationship of *E*. *bieneusi* genotypes

**Fig 1:**
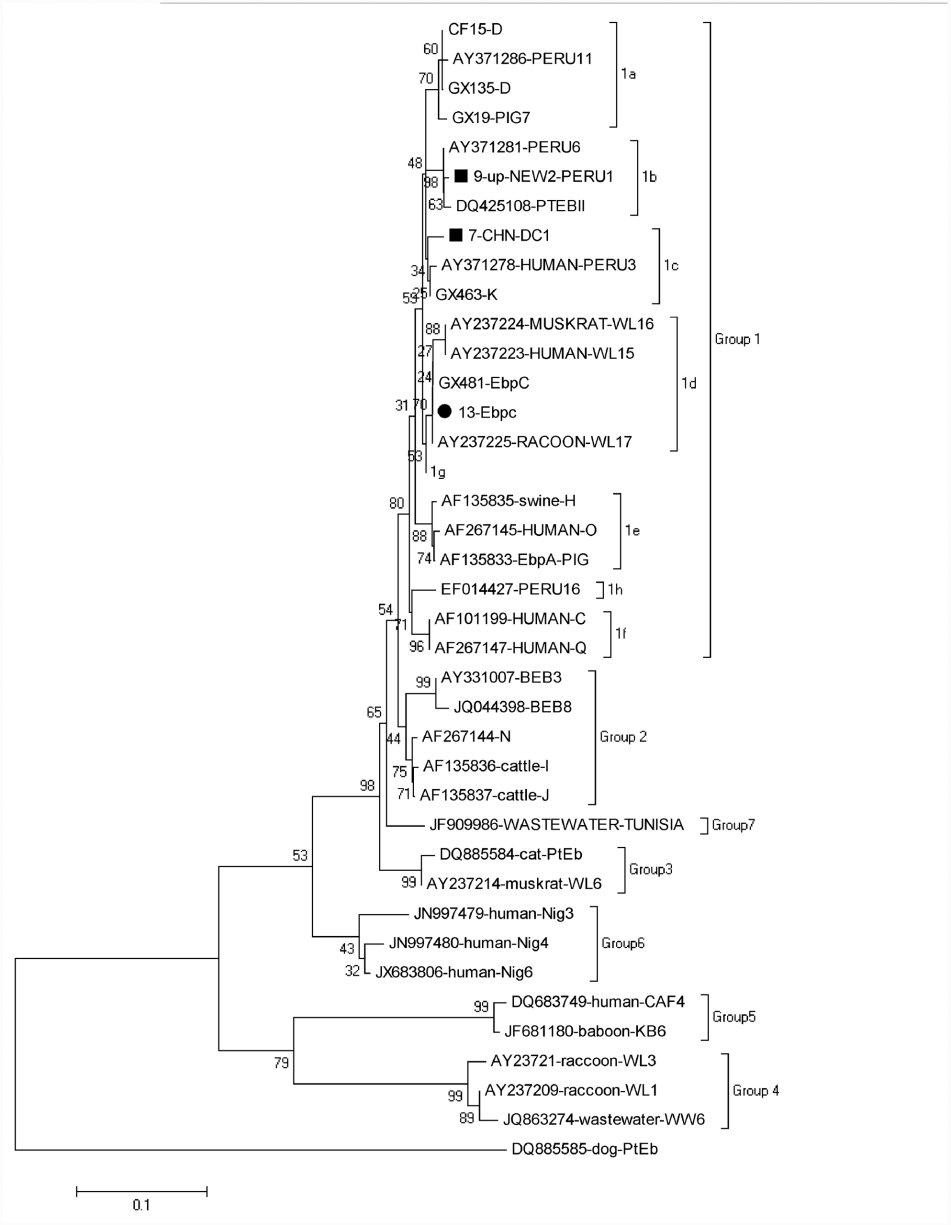
Phylogenetic relationship of *Enterocytozoon bieneusi* genotypes identified in the present study and other known genotypes deposited in GenBank was inferred by a neighbor-joining analysis of ITS sequences based on genetic distance by the Kimura two-parameter model. The numbers on the branches are percent bootstrapping values from 1,000 replicates. Each sequence is identified by its accession number, host origin, and genotype designation. The group terminology for the clusters is based on the work of Zhao et al. [26]. The squares and circles filled in black indicate novel and known genotypes identified in this study, respectively.

Based on a phylogentic analysis of a neighbor-joining tree of *E*. *bieneusi* genotypes, all the four genotypes (D, EbpC, CHN-F1 and CHN-R1) obtained here belonged to zoonotic group1, and they were further classed into subgroup 1a, 1d, 1c and 1b, respectively (Fig 1). Other genotype groups were not found in this study, and thus are not shown in Fig 1.

## Discussion

*E*. *bieneusi* is an emerging zoonotic pathogen. It has been confirmed to have a low host specificity and many domestic and wild animals may act as a reservoir host. Animals infected with *E*. *bieneusi* continue transmitting of this disease [20]. Despite the advances in exploring the genotypic and phylogenetic characteristics of *E*. *bieneusi* in a wide range of mammals and birds, few genetic studies have been documented in foxes, and none in raccoon dogs. *E*. *bieneusi* was found in 13.4% (9/67) foxes in the USA [19], while one of the seven wild foxes was detected positive for *E*. *bieneusi* in Spain [20]. Here, it was observed that infection rates of *E*. *bieneusi* in foxes (16.4%) and in raccoon dogs (4.1%) were lower than those reported in other animal hosts in the investigated areas, such as in pigs (89.5%), deer (31.9%), cattle (30.1%), sheep (22.5%) and goats (21.8%) [14,21,23,26]. However, it is difficult to explain the differences in infection rate of *E*. *bieneusi* between different hosts, for the rates of infection are influenced by many factors, such as immune competency of hosts, infection intensity of the pathogen, laboratory diagnosis of infections, sample size, animal management practices, as well as climate and geography.

An increasing body of information on genotyping of the ITS region of the ribosomal gene of *E*. *bieneusi* is now shedding light on the distributions of genotypes among humans and animals, and provides deeper understanding of the zoonotic nature of the pathogen as well as the transmission routes and sources. In the present study, four genotypes were identified out of 20 *E*. *bieneusi* isolates by analyzing the ITS region of the ribosomal sequences, comprising two known genotypes (D and EbpC) and two novel genotypes (CHN-F1 and CHN-R1). Here genotypes D, EbpC, CHN-F1 were found in foxes. In previous studies, four genotypes (D, Peru5, EbpC and WL15) were identified in nine wild fox-derived isolates of *E*. *bieneusi* in Maryland, USA [19]; genotype D was found in a wild fox positive for *E*. *bieneusi* in Spain [20]. Genotype D appeared to be common in foxes, and had a wide geographical distribution. The findings of the novel genotypes in foxes implied that these animals may harbor more genotypes of *E*. *bieneusi*. The present study identified genotypes D, CHN-R1 of *E*. *bieneusi* in raccoon dogs as a new host, replenishing molecular epidemiological data of *E*. *bieneusi* infection in animal species. Due to a small number of fox- and raccoon dog-derived *E*. *bieneusi* isolates, the true genotype constitutions of *E*. *bieneusi* in foxes and raccoon dogs need to be confirmed by more systematic epidemiological studies of *E*. *bieneusi* from these animals in the future.

Known genotypes D (syn. WL8, Peru9, pigEBITS9, PtEb VI, CEbC) and EbpC (syn. E, WL13, Peru4, WL17) are commonly described in humans, and have a wide host range and geographic distribution [4,6]. To date, genotype D has been found in over 15 animal species (cattle, pigs, sheep, dogs, cats, horses, macaques, gorillas, beavers, otters, muskrats, raccoons, and foxes as well as pigeons and falcons) from 24 countries [14,28]. Likewise genotype EbpC has been found in 10 animals (cattle, pigs, sheep, dogs, no human primates, beavers, otters, muskrats, raccoons, and foxes) from 12 countries [29]. In addition, genotypes D and EbpC have also been found in some water sources, including lake water, wastewater and drinking source water [8,11,30,31]. The facts above well establish the zoonotic nature and public health significance of the two genotypes. Thus, we should develop a better farm management system to prevent the occurrence of cross-transmission and re-infection of *E*. *bieneusi* between individuals in each farm. More importantly, measures must be taken to control environmental contamination and human infection of *E*. *bieneusi* spores.

In the phylogenetic tree of the ITS region sequences of *E*. *bieneusi* isolates, the two novel genotypes CHN-F1 and CHN-R1 belonged to the subgroup 1c and the subgroup 1b, respectively, suggesting the potential of zoonotic transmission. In fact, the genotypes CHN-F1 and CHN-R1 had only three and one base differences compared to human-pathogenic Type IV and Peru6 in group 1, respectively.

In conclusion, the present study confirms for the first time the occurrence of *E*. *bieneusi* in farmed blue foxes and raccoon dogs in China. The facts of genotypes D and EbpC commonly found in humans and animals, and the novel genotypes CHN-F1 and CHN-R1 falling into zoonotic group 1, suggest that foxes and raccoon dogs can serve as potential reservoir hosts for zoonotic *E*. *bieneusi* genotypes, and may constitute a risk for public health.

## Supporting Information

S1 Fig Geographical locations of farms involved in the present study in China. (DOCX)

S1 Table Information of *E*. *bieneusi* isolates. (DOCX)

S1 Text Basic information and geographical locations of animal farms. (DOCX)
