# Correction: Genotyping of *Enterocytozoon bieneusi* in Farmed Blue Foxes (*Alopex lagopus*) and Raccoon Dogs (*Nyctereutes procyonoides*) in China

**DOI:** 10.1371/journal.pone.0143992

**Published:** 2015-11-30

**Authors:** Wei Zhao, Weizhe Zhang, Ziyin Yang, Aiqin Liu, Longxian Zhang, Fengkun Yang, Rongjun Wang, Hong Ling

Fig 1 is incorrect in the published article. The authors have provided a corrected version here.

**Fig 1:**
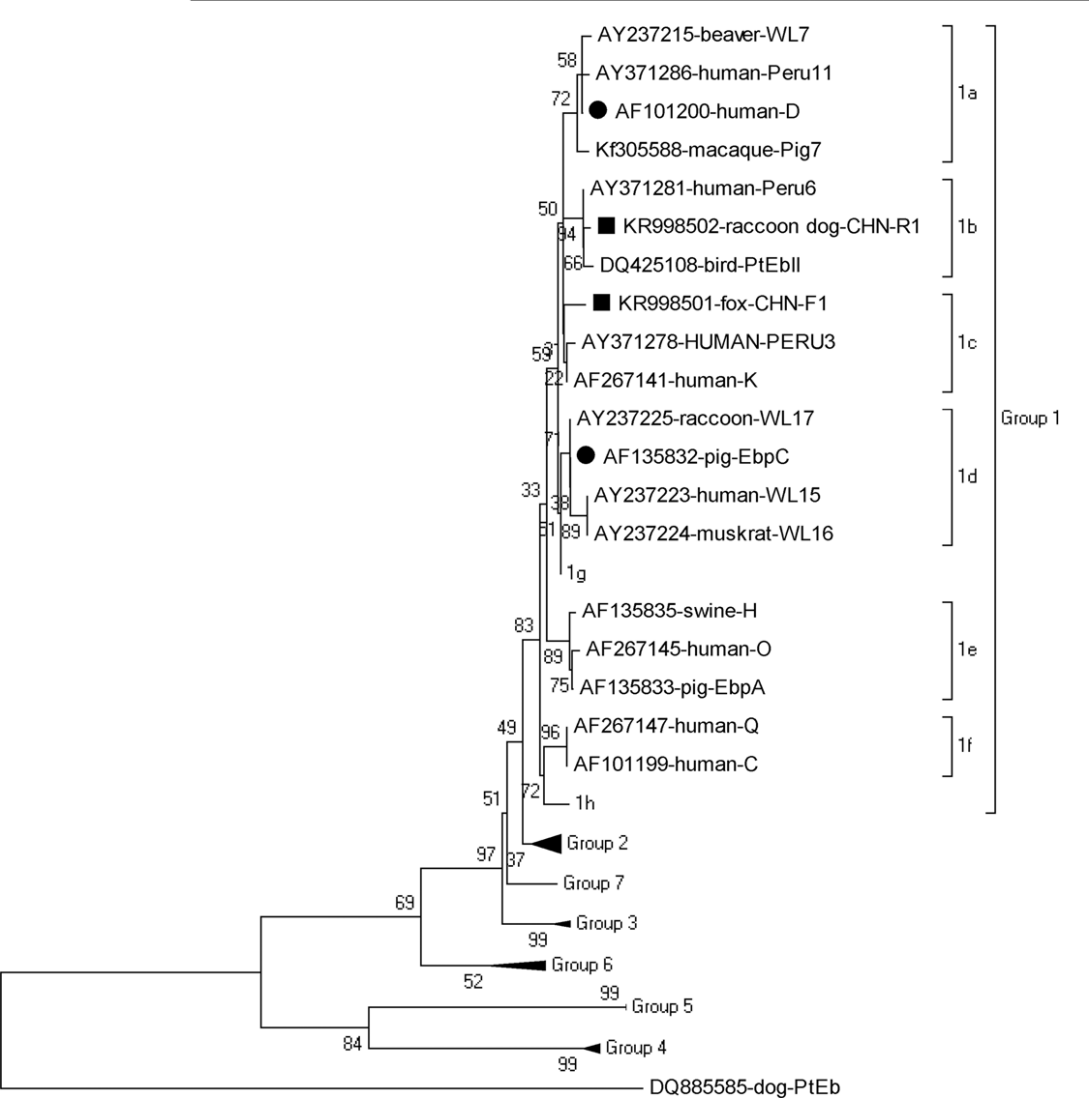
Phylogenetic relationship of *Enterocytozoon bieneusi* genotypes identified in the present study and other known genotypes deposited in GenBank was inferred by a neighbor-joining analysis of ITS sequences based on genetic distance by the Kimura two-parameter model. The numbers on the branches are percent bootstrapping values from 1,000 replicates. Each sequence is identified by its accession number, host origin, and genotype designation. The group terminology for the clusters is based on the work of Zhao et al. [26]. The squares and circles filled in black indicate novel and known genotypes identified in this study, respectively.
