# Supplementary material for: Genotyping of Enterocytozoon bieneusi in Farmed Blue Foxes (Alopex lagopus) and Raccoon Dogs (Nyctereutes procyonoides) in China
Source: PLoS One. 2015 Nov 6;10(11):e0142611. doi: 10.1371/journal.pone.0142611 (PMC4636423; doi:10.1371/journal.pone.0142611)
Supplement: S1 Fig — (DOCX) [file pone.0142611.s001.docx]

**S1 Fig.** **Geographical locations** **of farms involved in the present study in China.** HLJ=Heilongjiang Province; JL=Jilin Province. SH=Suihua City; BY=Bayan County; CC=Changchun City.

**
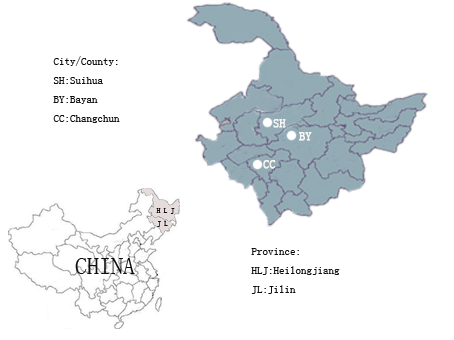
**
