# Supplementary material for: Genotyping of Enterocytozoon bieneusi in Farmed Blue Foxes (Alopex lagopus) and Raccoon Dogs (Nyctereutes procyonoides) in China
Source: PLoS One. 2015 Nov 6;10(11):e0142611. doi: 10.1371/journal.pone.0142611 (PMC4636423; doi:10.1371/journal.pone.0142611)
Supplement: S1 Table — (DOCX) [file pone.0142611.s002.docx]

**S1 Table. Information of *E. bieneusi* isolates**

| **Farm (host)** | **Specimen code** | **Location (province)** | **Genotype** |
| --- | --- | --- | --- |
| Farm 1 (blue fox) | BF-1 | Bayan (Heilongjiang) | D |
|  | BF-16 | Bayan (Heilongjiang) | D |
|  | BF-19 | Bayan (Heilongjiang) | D |
|  | BF-30 | Bayan (Heilongjiang) | D |
|  | BF-44 | Bayan (Heilongjiang) | D |
|  | BF-48 | Bayan (Heilongjiang) | D |
| Farm 2 (blue fox) | SF-4 | Suihua (Heilongjiang) | EbpC |
|  | SF-11 | Suihua (Heilongjiang) | EbpC |
|  | SF-12 | Suihua (Heilongjiang) | EbpC |
|  | SF-14 | Suihua (Heilongjiang) | EbpC |
|  | SF-20 | Suihua (Heilongjiang) | CHN-F1 ( KR998501) |
|  | SF-23 | Suihua (Heilongjiang) | EbpC |
| Farm 3 (blue fox) | CF1-4 | Changchun (Jilin ) | D |
|  | CF1-11 | Changchun (Jilin ) | D |
| Farm 4 (blue fox) | CF2-3 | Changchun (Jilin ) | D |
|  | CF2-10 | Changchun (Jilin ) | D |
|  | CF2-16 | Changchun (Jilin ) | D |
|  | CF2-20 | Changchun (Jilin ) | D |
| Farm 5 (raccoon dog) | BR-36 | Bayan (Heilongjiang) | CHN-R1 ( KR998502) |
|  | BR-43 | Bayan (Heilongjiang) | D |

Note: BF= Foxes in Bayan; SF= Foxes in Suihua; CF1= Foxes in the first farm in Changchun; CF2= Foxes in the second farm in Changchun; BR= Raccoon dogs in Bayan. KR998501 and KR998502 are accession numbers of genotypes CHN-F1 and CHN-R1 in GenBank.
