# Supplementary material for: Genotyping of Enterocytozoon bieneusi in Farmed Blue Foxes (Alopex lagopus) and Raccoon Dogs (Nyctereutes procyonoides) in China
Source: PLoS One. 2015 Nov 6;10(11):e0142611. doi: 10.1371/journal.pone.0142611 (PMC4636423; doi:10.1371/journal.pone.0142611)
Supplement: S1 Text — (DOCX) [file pone.0142611.s003.docx]

**S1 Text. Basic information and geographical locations of animal farms**

Farm 1 and Farm 5 belong to Huaqiang Fur Breeding Base located in Bayan County (46º08′N, 127º04′E) in Heilongjiang Province, which are accredited by branch of fur animal breeding of Heilongjiang Animal Agriculture Association. Foxes (mainly blue foxes) and raccoon dogs as well as rex rabbits are bred separately in the base. In our study only approximately 10% blue foxes and raccoon dogs in two farms were involved.

Farm 2 in Mingshui County (47º18′N, 125º09′E) in Suihua City of Heilongjiang Province, and Farm 3 and Farm 4 in Kuancheng District (43º92′N, 125º32′E) of Changchun City of Jilin Province are individual farms, which are accredited by Suihua and Changchun animal husbandry and veterinary bureaus, respectively. Only blue foxes are housed on each the three small private farms. Also, approximately 10% animals were randomly collected to participate in our investigation of *E. bieneusi*.
